# Translational Control of the HIV Unspliced Genomic RNA

**DOI:** 10.3390/v7082822

**Published:** 2015-08-04

**Authors:** Bárbara Rojas-Araya, Théophile Ohlmann, Ricardo Soto-Rifo

**Affiliations:** 1 Molecular and Cellular Virology Laboratory, Program of Virology, Institute of Biomedical Sciences, Faculty of Medicine, University of Chile, Independencia 834100, Santiago, Chile; E-Mail: barbara.rojas.araya@gmail.com; 2 CIRI, International Center for Infectiology Research, Université de Lyon, Lyon 69007, France; 3 Inserm, U1111, Lyon 69007, France; 4 Ecole Normale Supérieure de Lyon, Lyon 69007, France; 5 Université Lyon 1, Centre International de Recherche en Infectiologie, Lyon 69007, France; 6 CNRS, UMR5308, Lyon 69007, France

**Keywords:** HIV-1, Rev, nuclear export, DDX3, cap-dependent translation, IRES

## Abstract

Post-transcriptional control in both HIV-1 and HIV-2 is a highly regulated process that commences in the nucleus of the host infected cell and finishes by the expression of viral proteins in the cytoplasm. Expression of the unspliced genomic RNA is particularly controlled at the level of RNA splicing, export, and translation. It appears increasingly obvious that all these steps are interconnected and they result in the building of a viral ribonucleoprotein complex (RNP) that must be efficiently translated in the cytosolic compartment. This review summarizes our knowledge about the genesis, localization, and expression of this viral RNP.

## 1. Introduction

Human Immunodeficiency virus type-1 (HIV-1) and type-2 (HIV-2) belong to the Lentivirus genus of the *Retroviridae* family and are the etiological agents of the Acquired Immunodeficiency Syndrome (AIDS) in humans [1]. Both viruses primarily infect cells of the immune system that express the CD4 receptor and one of the chemokine receptors CCR5 or CXCR4 that act as co-receptors for viral entry. The HIV replication cycle begins with the interactions between the surface glycoprotein gp120 with CD4 and one of the co-receptors in a process that induces conformational changes allowing insertion of the viral transmembrane protein gp41 in the host cell membrane to trigger fusion of both membranes and entry of the viral capsid into the host cell cytoplasm. Then, the positive single stranded RNA genome is converted into double stranded DNA by the virally encoded reverse transcriptase, which is located in the capsid. In association with viral and cellular proteins, viral DNA forms the so-called pre-integration complex (PIC), which is imported to the host cell nucleus in an active process orchestrated by the viral proteins capsid and integrase [2]. The latter then catalyzes integration of viral DNA into the host cell genome to establish what is known as the proviral state. Once integrated, the provirus can remain latent or undergo efficient gene expression in order to continue with late steps of the replication cycle. The full-length unspliced genomic RNA (hence referred as unspliced mRNA) has a dual function as it is both used as mRNA for the synthesis of Gag and Gag-Pol precursors and the genome that is incorporated into the viral particles. The structural protein Gag drives both packaging of the genomic RNA and assembly of newly synthesized viral particles, which will be maturated by the viral protease allowing initiation of a new replication cycle.

**Figure 1:**
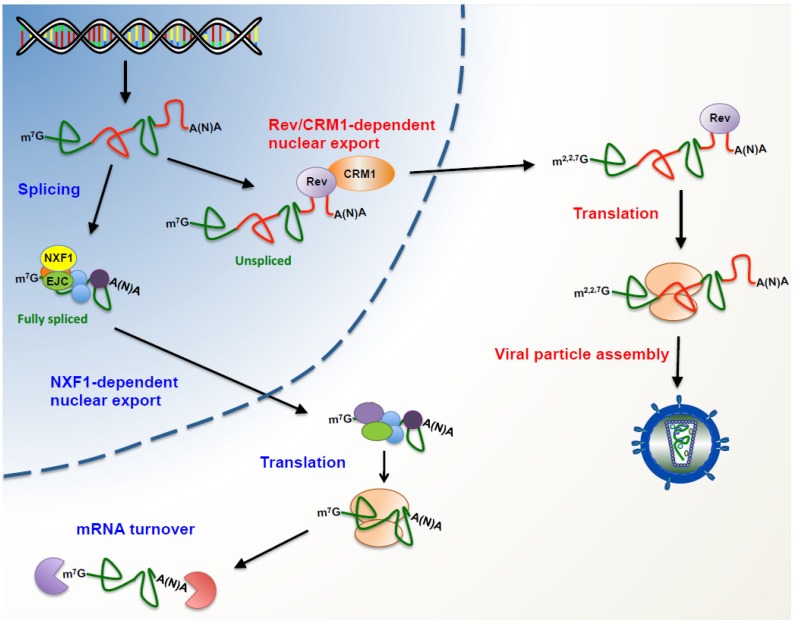
Post-transcriptional control on human immunodeficiency virus (HIV). Upon transcription, the capped and polyadenylated full-length genomic RNA is used as a template for the host mRNA processing machinery in order to generate fully spliced and partially spliced transcripts (partially spliced transcripts have been omitted for simplicity). In the nucleus, fully spliced transcripts form a classical messenger ribonucleoprotein complex (mRNP) together with host proteins such as the exon junction complex (EJC) and the mRNA export factor NXF1. In the cytoplasm, fully spliced mRNAs recruit the host translational apparatus for protein synthesis and later they are degraded by the mRNA turnover machinery. In the presence of the viral protein Rev, the unspliced genomic RNA (and partially spliced mRNAs) reaches the cytoplasm through the CRM1-dependent pathway avoiding the host cell surveillance mechanisms. During this journey to the cytoplasm, the unspliced genomic RNA forms a unique mRNP that favors its association with the host translational machinery. In contrast to the fully spliced transcripts, the unspliced genomic RNA does not undergo turnover as it is incorporated into viral particles.

HIV gene expression relies on the host for transcription, RNA processing, nuclear export and translation, a series of complex processes that are assisted by at least, two major viral regulators namely Tat and Rev. HIV transcription relies both on the promoter sequences present in the viral 5′ long-terminal repeat (5′-LTR) region and the *trans*-activator viral protein Tat, which acts together with host cellular proteins including the RNA polymerase II and the pTEFb transcription factor [3,4,5,6,7]. Transcription from the provirus results in expression of the full-length unspliced mRNA, which is 9-kb long and encodes structural and enzymatic proteins (Gag and Gag-Pol). However, the presence of multiple splice donor and acceptor sites within the full-length mRNA supports alternative splicing which results in the generation of a complex pattern of viral mRNAs harboring the open reading frames of Vif, Vpr, Vpu/Env, Tat, Rev and Nef, which differ in their 5′ untranslated regions (5′-UTR) [8]. These transcripts are both incompletely (4-kb) and completely spliced (2-kb) and are used for expression of all remaining viral proteins. Several of these completely spliced transcripts coding for Tat, Rev, and Nef are produced during the early steps of infection [9,10,11]. Later on, the full-length unspliced mRNA together with further different 4-kb transcripts coding for Env/Vpu, Vif, Vpr, and Tat are then generated, exported and translated in the cytoplasm [9,10,11,12]. All these RNA processing events generate different viral mRNP complexes that will differ in the routes used to reach the host translational machinery. As such, while completely spliced transcripts are exported by the canonical nuclear export pathway, the unspliced and the 4-kb incompletely spliced transcripts require the binding of the virally encoded protein Rev to the *cis*-acting RNA element called the Rev responsive element (RRE) present in all of these intron-containing transcripts; this allows their export through the CRM1 pathway [13,14,15,16,17,18,19,20] (Figure 1).

As mentioned above, in addition to its nuclear function as a pre-mRNA template for the generation of the 2 and 4-kb transcripts, the 9-kb full-length unspliced mRNA plays two additional roles in the cytoplasm by serving both as a mRNA for viral protein production and as the packaged genome (Figure 1). In order to combine these different functions, the unspliced mRNA needs to overcome several structural and functional constraints that could affect cellular post-transcriptional events such as nuclear export and translation. In this review, we focus on how the virus has evolved to combine the building of a complex and specific mRNP on its mRNAs ensuring proper viral gene expression.

## 2. Reaching the Cytoplasm Avoiding Surveillance Mechanisms

HIV-1 transcripts are synthesized by the RNA polymerase II and, consequently, are capped and polyadenylated by the host machinery [21,22,23,24]. As described above, many different viral mRNA species can be found in infected cells with at least four different 5′- and eight different 3′-splice sites being used during pre-mRNA processing [9,10,11]. However, the cellular splicing machinery must be inefficient in the usage of viral splice sites in order to ensure that appropriate pools of each subset of viral mRNAs can be produced in the nucleus [25,26,27]. The vast majority of cellular mRNAs are usually spliced to completion and thus all introns are removed during splicing [28]. This is the case for the viral 2-kb transcripts that are completely processed and can be exported to the cytoplasm through the nuclear export factor NXF1 [29,30,31]. However, nuclear export of mRNAs that harbor functional introns is quite unusual and they are often retained in the nucleus by the interaction with splicing factors until they are either spliced to completion or degraded [32,33,34]. In addition, viral intron-containing transcripts cannot be exported through the NXF1-dependent pathway due to surveillance mechanism ruled by, amongst others, the cellular protein Tpr [35,36,37]. As such, the 4-kb incompletely spliced and the 9-kb unspliced transcripts are retained and degraded in the host cell nucleus unless the viral protein Rev is present [25,38].

Rev is synthesized from a 2-kb completely spliced transcript and is essential for virus replication [39]. Although the step of the replication cycle in which Rev activity is the most important has been the subject of some controversy [39,40,41], there is no doubt that synthesis of the viral structural proteins Gag and Gag-Pol from the unspliced mRNA is dramatically reduced in the absence of Rev. Rev is a phosphoprotein of approximately 18-kDa that constantly shuttles between the nucleus and the cytoplasm but accumulates in the nucleus [39]. The N-terminal domain of the protein contains an arginine-rich motif that serves both as a nuclear localization signal (NLS) and as an RNA-binding domain (RBD) [39,42,43,44,45,46,47,48,49,50]. While the NLS allows recognition and nuclear import of Rev by Importin-β, the RBD allows the interaction with the Rev Responsive Element (RRE) which is present exclusively in the incompletely spliced and unspliced viral transcripts as it is located within the *env* gene [19,39,51]. The arginine-rich sequence is flanked from both sides by less defined sequences required for oligomerization [39,49,50]. The C-terminal domain contains the leucine-rich nuclear export signal (NES) that allows the interaction and nuclear export of the Rev-RRE complex with the karyopherin CRM1 (Chromosome maintenance-1) bound to Ran-GTP [16,52,53,54,55]. Recent structural studies have revealed that once bound to the RRE, the Rev protein oligomerizes in order to promote nuclear export [49,56] while CRM1 forms a dimer that favors nuclear export of the Rev-RRE complex [57]. Moreover, it was recently shown that Rev can interact with the nuclear cap-binding complex (CBC) component CBP80 and block NXF1 recruitment in order to specifically enter the nuclear export pathway through CRM1 [58]. In addition to CRM1-RanGTP and CBP80, Rev recruits several host proteins including eIF5A, hRIP, DDX3, DDX1, and Sam68 to promote nuclear export [59]. Thus, by using this alternative pathway, the viral protein Rev ensures the cytoplasmic accumulation of intron-containing transcripts and avoids NXF1-associated quality control mechanisms. This explains that despite the presence of introns, viral transcripts that do not undergo complete splicing are not substrates for non-sense mediated decay (NMD) [60,61].

After completion of their journey from the nucleus and through the nuclear pores, the viral transcripts must compete with cellular mRNAs in the cytoplasm to recruit the host translational machinery. In mammals, ribosome recruitment onto the mRNA occurs by two main mechanisms: the cap-dependent and the internal ribosome entry sites (IRES)-driven mechanisms [62,63] and HIV-1 has evolved strategies to use both [64].

## 3. An Overview on mRNA Translation Initiation in Eukaryotes

The vast majority of cellular mRNAs recruit ribosomes through a cap-dependent translation initiation mechanism. This process sequentially involves: (i) formation of a 43S pre-initiation complex; (ii) cap structure recognition and loading of the 43S pre-initiation complex onto the mRNA; (iii) ribosomal scanning of the 5′-UTR; (iv) initiation codon recognition and (v) joining of the 60S ribosomal subunit [62]. The 43S pre-initiation complex is composed of a recycled 40S small ribosomal subunit, an eIF2-GTP-tRNAi ternary complex (TC), eIF3, eIF1, eIF1A and probably eIF5 [62]. At the 5′ end of the mRNA, the eIF4F holoenzyme binds to the cap-structure and unwinds local RNA structures assisted by eIF4B or eIF4H creating the landing pad for the 43S pre-initiation complex. The eIF4F multimeric complex is composed of the cap-binding protein eIF4E, the RNA helicase eIF4A, and the scaffold protein eIF4G [65,66]. eIF4E exhibits high affinity for the cap structure and interacts with eIF4G to mediate cap-dependent translation initiation by promoting assembly of eIF4F onto the capped mRNA. The DEAD-box protein eIF4A is an RNA helicase with ATP-dependent RNA unwinding activity [67,68]. Although the intrinsic helicase activity of eIF4A is weak, its inclusion into the eIF4F complex together with the binding of eIF4B and the related factor eIF4H strongly stimulates its enzymatic activity [69]. As mentioned above, the eIF4G scaffold protein associates with eIF4E and eIF4A to form the eIF4F holoenzyme that binds to the 5′ end of capped mRNAs [70,71,72,73]. By further interacting with eIF3, eIF4G promotes attachment of the 43S pre-initiation complex onto the transcript to allow formation of a 48S pre-initiation complex [72,74,75,76,77,78]. Once attached, this complex immediately starts scanning in a 5′ to 3′ direction from the cap structure until it reaches an initiation codon, which often corresponds to the first AUG codon [79,80,81]. The ribosomal scanning model proposes that the translation initiation complex unwinds secondary structures present in the 5′-UTR and moves in the 5′ to 3′ direction in an ATP-dependent manner [82,83]. Thus, in addition to their role in 43S pre-initiation complex attachment, eIF4G, eIF4A, eIF4B (or eIF4H) also assist the scanning process [83,84]. Although the RNA helicase eIF4A and its associated factors eIF4B/eIF4H can support the unwinding process of the scanning pre-initiation complexes, it has been recently shown that additional RNA helicases can also be recruited [85]. As such, the related DExH box protein 29 (DHX29) binds the 40S small ribosomal subunits while RNA helicase A binds selected mRNAs and both are required for efficient scanning of mRNAs containing highly structured 5′-UTRs [86,87,88].

An alternative model of translation initiation has been described for mRNAs that harbor specific RNA sequences termed Internal Ribosome Entry Sites (IRES). These sequences are generally present in the 5′-UTR of the mRNA whose function is to recruit ribosomes for translation initiation in a cap-independent manner. IRES elements were first discovered in viral RNA genomes more than 25 years ago with the studies of picornavirus translation [89,90] and have now been characterized in many viral mRNAs including HCV, Pestiviruses, and Retroviruses [63]. Although IRES elements have also been described in near 100 cellular mRNAs their existence remains controversial mainly by the lack of essential controls discarding cryptic promoters and/or alternative splicing during the characterization process [91].

IRES elements promote the direct binding of the 43S pre-initiation complex and associated factors to the mRNA. However, the precise mechanism of IRES-mediated translation initiation is not completely understood. Although a classification of IRES elements by structural criteria is not possible due to the lack of any conserved sequence, viral IRES elements can be grouped based on a mechanistic and functional point of view involving: (i) the way by which the 43S pre-initiation complexes is recruited, e.g., whether it is assisted or not by eIFs; and (ii) the site where the 43S pre-initiation complex is positioned onto the mRNA, which can be close to the initiation codon or if it involves an additional step of scanning. Moreover, IRES elements can also be characterized by the requirement of diverse cellular accessory proteins denominated IRES *trans*-acting factors (ITAFs) for proper function.

IRES elements allow the selective translation of viral mRNAs under conditions in which global host translation is compromised. When faced by several stresses (such as viral infections, hypoxia, or heat shock) or particular cellular conditions (such as mitosis or apoptosis), Eukaryotic cells often respond by reducing the global rates of translation [92]. However, a significant fraction of cellular mRNAs was shown to remain associated to polysomes [93] and several of these are IRES-containing transcripts. This shows that the presence of the IRES element allows mRNAs to be translated under unfavorable conditions in which cap-dependent translation is slowed down or arrested [94,95,96,97,98].

## 4. Recruiting the Host Translational Machinery onto the Unspliced HIV Genomic RNA

The unspliced mRNA harbors a long (5′-UTR) organized in several RNA structures involved in many steps of the replication cycle [3,99,100,101,102,103]. Given the structure and complexity of the 5'-UTR, the mechanism by which translation initiation takes place on the HIV-1 genomic RNA has been the subject of debate for several years [104]. Indeed, it was initially shown that sequences derived from the 5′-UTR were inhibitory for translation [105,106,107,108,109]. Particularly, cell-free *in vitro* translation assays and *ex vivo* experiments using reporter genes suggested that the presence and folding of the TAR RNA motif, which is located at the very 5′ end of the viral transcripts, exerted a negative effect on protein synthesis both by impeding ribosome recruitment and by activating the kinase PKR [105,106,107,108,110,111]. However, despite this incompatibility with ribosome recruitment by a cap-dependent ribosomal scanning mechanism, an IRES-driven mechanism on HIV-1 transcripts was rapidly discarded indicating that cap-dependent translation initiation was the major mechanism for ribosome recruitment [112].

## 5. Identification of a Cell Cycle-Dependent IRES

As mentioned above, initial attempts to identify sequences within viral 5′-UTR supporting IRES activity failed and the cap-dependent ribosomal scanning was proposed as the only mechanism to drive Gag synthesis [112]. However, a more detailed study revealed that an IRES element was indeed present within the 5′-UTR of the HIV-1 unspliced mRNA [113]. This IRES element was mapped to nucleotides 104 to 336 where it spans the primer-binding site (PBS), the dimerization site (DIS), the major splice donor (SD) and RNA motifs that are critical for encapsidation [113]. Interestingly, this IRES element was shown to be activated during the G2/M phase of the cell cycle [113]. This peculiarity not only explained why previous studies failed to detect IRES activity but also emphasized the physiological relevance that the use of an alternative mechanism of ribosome recruitment could have during viral replication. Indeed, during HIV-1 and other lentiviral infections, the viral protein Vpr induces a cell cycle arrest at the G2/M phase [114,115,116,117]. Although the G2/M phase is characterized by a strong inhibition of cap-dependent protein synthesis [95,118,119], HIV-1 viral gene expression was shown to continue during this phase of the cell cycle [120,121,122]. Although IRES elements were demonstrated to be able to drive efficiently protein synthesis in G2/M [96], other authors have proposed another alternative in which the translation of the HIV-1 unspliced mRNA was rather conducted by a eIF4E-independent, CBC-driven, cap-dependent mechanism during the G2/M arrest induced by Vpr [122]. Nevertheless, the ability of the 5′-UTR of HIV-1 transcripts to drive IRES-driven translation has now been evidenced by several groups in different experimental contexts and on different HIV-1 prototype strains [123,124,125,126,127,128,129,130]. Therefore, it is conceivable that the HIV-1 genomic RNA can use both strategies depending on some physiological conditions that remain to be found. Moreover, similar to poliovirus, the HIV-1 and HIV-2 proteases were shown to process translation initiation factors eIF4GI and PABP *in vitro* and *ex vivo* leading to the inhibition of cap-dependent ribosomal scanning with modest impact on viral unspliced mRNA translation [70,131,132,133,134]. However, processing of eIF4GI and PABP during viral infection was rather modest and occurred late during infection [131] and thus, the significance of these events in the course of viral replication remains to be demonstrated.

Protein synthesis from the HIV-1 and HIV-2 unspliced mRNAs presents an additional layer of complexity as IRES elements have also been characterized within the Gag coding region [64,135]. By using the HIV-1 Gag ORF lacking the viral 5′-UTR it was shown that this region was able to drive synthesis of full-length p55 Gag and a novel 40-kDa N-terminally truncated isoform of Gag (p40) initiated at an internal *in frame* AUG codon [136]. The presence of IRES elements downstream to the authentic initiation codon and the synthesis of N-terminally truncated isoforms of Gag were also characterized in other related lentiviruses such as HIV-2, SIV, and FIV indicating that the conservation of the mechanism is a common feature of the genus and could be important for replication [135,137,138,139]. In HIV-1 and HIV-2, these Gag isoforms are incorporated into viral particles despite the lack of a myristoylation site at their N-terminus, probably by protein-protein interactions with the full length Gag polyprotein; this suggests a role for these truncated isoforms in the replication cycle [136,138]. Although the molecular mechanisms controlling this process in the HIV-1 unspliced mRNA are not completely understood, an *in vitro* study revealed that the different modes of ribosome recruitment have different levels of requirements for eIF4F [140]. In the case of the HIV-2, it was shown that three IRES elements within the Gag coding region were able to directly recruit three independent 43S pre-initiation complexes [138,141,142,143].

## 6. Translation by a Cap-Dependent Mechanism

More recently, by using synthetic constructs Berkhout and co-workers demonstrated that cap-dependent ribosomal scanning occurs throughout the 5′-UTR of the HIV-1 unspliced mRNA [144]. Using similar approaches, other groups including ours demonstrated that the cap-dependent mechanism of translation initiation occurs both *in vitro* and *ex vivo* [109,121]. The ability of the 43S pre-initiation complex to scan through the highly structured 5′-UTR could be explained by the recruitment of the helicase RHA, which was shown to promote polysome association of the unspliced mRNA by interacting with a post-transcriptional control element (PCE) located at the 5′-UTR [88,145]. Although it is thought that RHA helicase activity contributes to the unwinding of secondary structures during ribosomal scanning, the involvement of other RNA helicases such as DHX29 has not yet been investigated and thus, cannot be discarded.

**Figure 2:**
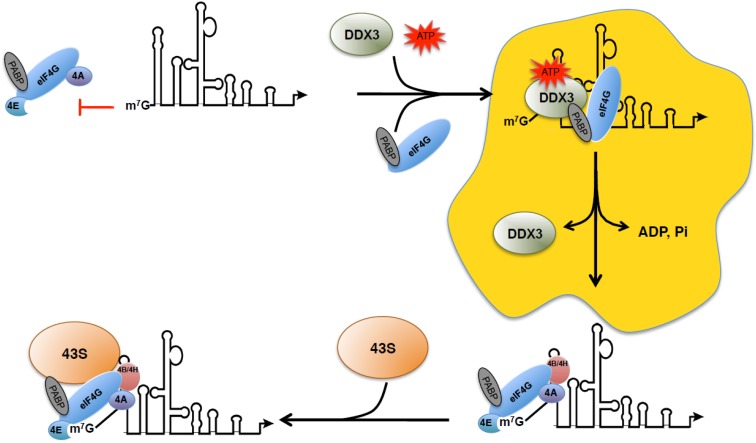
DDX3-mediated translation of the HIV-1 genomic RNA. In the absence of DDX3, the TAR RNA motif impedes binding of the eIF4F holoenzyme to the cap. Thus, DDX3 binds the viral 5′-UTR to nucleate formation of a pre-translation initiation complex that involves ATP-dependent unwinding of TAR and specific recruitment of translation initiation factors eIF4GI and PABP (and probably other unidentified cellular proteins). TAR unwinding renders the cap accessible for eIF4F binding and subsequent recruitment of the 43S pre-initiation complex. It is possible that such a pre-translation initiation step driven by DDX3 occurs compartmentalized in RNA granules (in yellow).

While the involvement of RHA shed light on how the 43S pre-initiation complex moves along the highly structured viral 5′-UTR, it was still unclear how the cap-structure could be recognized by the eIF4F complex in the presence of the TAR structure. Indeed, the 5′ end cap moiety of all HIV-1 transcripts is base-paired and embedded within the basis of the stem of the TAR RNA motif and thus, is likely to be inaccessible for the binding of the eIF4F complex and the ribosomal 43S subunit. Surprisingly, although the presence of TAR was shown to strongly interfere with translation initiation in the rabbit reticulocytes lysate (RRL), this was not the case in constructs expressed in living cells [109]. These data suggested that some specific host factor(s) that are absent or in limited concentration in the RRL can be used to overcome the structural constraint imposed by TAR. A likely candidate was found amongst one of the Rev cofactors namely the RNA helicase DDX3 [146]. DDX3 belongs to the DEAD-box family of proteins whose prototype member is the initiation factor eIF4A [147]. DEAD-box proteins are ATP-dependent RNA helicases that play pleiotropic functions within the cell by participating in all steps of RNA metabolism [148]. These proteins are thought to participate in RNA:RNA and RNA:protein remodeling or to act as RNA clamps for the assembly of large macromolecular complexes [148]. DDX3 was first proposed to be a host factor involved in Rev-dependent nuclear export [146]. By using a full-length reporter proviral DNA and viral infection, we were also able to show that DDX3 was required for translation of the unspliced genomic RNA both in HeLa and T-cells and this function required the ATP binding and ATPase activity of the enzyme [149,150]. We also reported that the molecular target for DDX3 was actually the TAR RNA motif, an observation recently validated by another group [151]. Interestingly, we observed that DDX3 was required to unwind TAR in cells and this functional interaction was necessary when the latter was at its original location (e.g., at the 5′ end of the HIV-1 transcript) but the dependence in DDX3 was abolished when the TAR motif was preceded by an unstructured spacer sequence [149]. These data suggested that DDX3 binds and unwinds TAR during a pre-translation initiation step that is necessary to remodel secondary structures in order to render the cap moiety accessible to the eIF4F holenzyme and the 43S complex [149] (Figure 2). In agreement with this, we could also show that DDX3 was bound to, at least, two additional and specific sites within the 5′-UTR of the HIV-1 genomic RNA [149]. These sites were located exclusively on RNA single stranded regions and could correspond to loading platforms for DDX3 as had been previously suggested [148]. In addition, an interaction between DDX3 and translation initiation factors eIF4GI and PABPC1 was also evidenced by biochemical assays as well as confocal microscopy [149]. Interestingly, we observed that the complex formed between the unspliced HIV-1 mRNA, DDX3, eIF4GI, and PABPC1 was assembled in localized cytoplasmic granules that resembled but were different from stress granules as they lacked the eIF4F components eIF4E, eIF4A, eIF4B, and the CBC component CBP80 [150] (Figure 2).

Another intriguing feature of the unspliced HIV-1 mRNA that could also influence its translation is the presence of a trimethylguanosine (TMG) cap structure [152]. A few years ago, the peroxisome proliferator-activated receptor-interacting protein with methyltransferase domain (PIMT) (the human homolog of the yeast cap hypermethylase TGS1) was shown to interact with Rev and this resulted in the hypermethylation of the 5′ cap structure of the HIV-1 unspliced mRNA [152]. It has been known for quite some time that TMG-capped mRNAs present reduced translational rates *in vitro* [153]. However, in the case of the HIV-1 genomic RNA, the latter is efficiently used for viral protein production and trimethylation of its cap was shown to be required in this process although the molecular mechanism underlying was not elucidated [152]. In light of our data showing the presence of a pre-initiation complex composed of DDX3/eIF4G/PABP, but lacking any of the major cap binding proteins CBC and eIF4E [150], a further investigation into the role of a TMG cap would be interesting. Indeed, as the affinity of the CBC and eIF4E for the TMG cap is largely reduced compared to the classical m^7^G monomethylated cap [154], this could explain the exclusion of both eIF4E and CBC from this pre-initiation complex and could suggest that other TMG-bound cellular proteins may be recruited for initiation of HIV-1 unspliced mRNA translation.

## 7. Assembly of Unspliced mRNA-Containing Granules

Cellular mRNAs are in a dynamic equilibrium between polysomes and cytoplasmic granules such as stress granules and p-bodies [155,156]. While stress granules are sites of triage for mRNAs stalled in translation initiation as a response to cellular stress, p-bodies are sites intimately related to the mRNA decay machinery [155,156]. Both structures and/or some of their components have been shown to play pivotal roles during replication of several viruses and thus, it is not surprising that viruses have evolved different strategies to manipulate the assembly/disassembly of mRNA granules [157,158,159].

Although it was first proposed that HIV-1 translation could be negatively regulated by some components of p-bodies including APOBEC3G [160,161], there is new evidence showing that HIV-1 replication induces the disassembly of p-bodies [162]. Moreover, it was also recently shown that APOBEC3G activity on HIV-1 replication was independent of p-bodies [163] and that p-bodies components such as DDX6 and Argonaute 2 were rather involved in viral particle assembly independent of RNA packaging [164]. Therefore, further work is necessary to clarify the role of p-bodies in HIV-1 unspliced mRNA metabolism.

**Figure 3:**
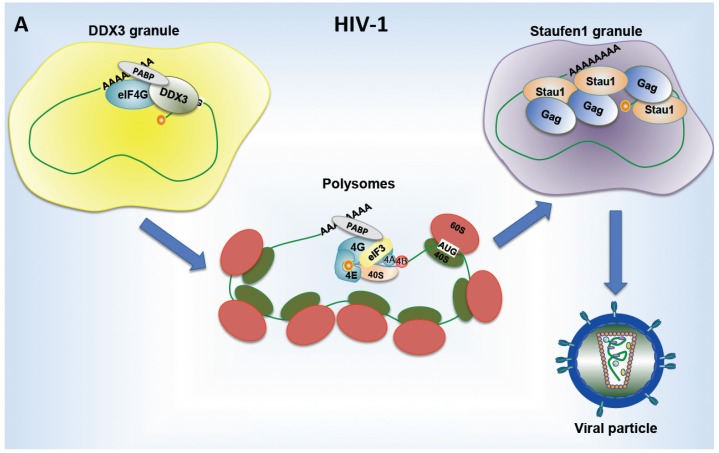

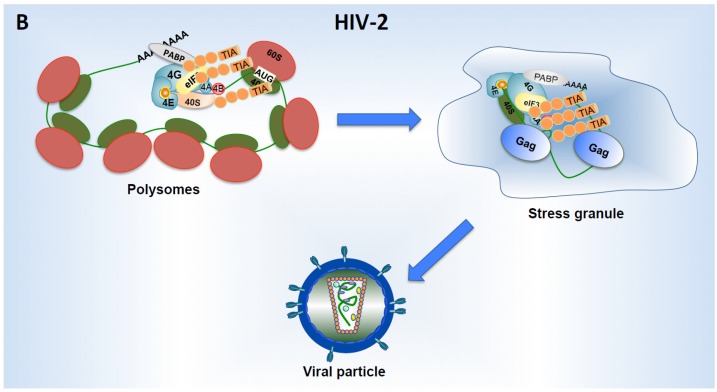
RNA granules assembled during HIV replication. (**A**) Polysome association of HIV-1 unspliced mRNA requires its previous assembly in DDX3-dependent granules together with eIF4GI, PABPC1 and probably other, yet, unidentified cellular proteins. Once translated, unspliced mRNA associates with the dsRNA-binding protein Staufen1 and the viral protein Gag in order to form another specific RNA granule (Staufen1 granule), which is required for viral particle assembly. This dynamic assembly of different RNA granules allows HIV-1 to coordinate genomic RNA translation and packaging; (**B**) The HIV-2 unspliced mRNA recruits the stress granule assembly factor TIAR to form a specific viral mRNP that accumulates in stress granules in the absence of active translation. The viral protein Gag also accumulates in stress granules suggesting that the transition from translation to RNA packaging could occur in these structures.

Interestingly, HIV-1 and HIV-2 have evolved completely opposite strategies to modulate and control the assembly of stress granules. As such, it was shown that HIV-1 has the ability to interfere with stress granule assembly induced by different types of stresses [162,165]. Indeed, the authors showed that the HIV-1 Gag protein has the ability to interfere with stress granules assembly through a direct interaction with eEF2 and G3BP1, two key factors required for assembly of these cytoplasmic structures [165]. Thus, it is possible that by doing so, the HIV-1 unspliced mRNA promotes the assembly of a pre-initiation complex with DDX3 and subset of eIFs in order to enter in translation initiation and associate with polysomes [150] (Figure 3A). Then, the HIV-1 unspliced mRNA is assembled into a Staufen1-dependent mRNP, which also contains the viral protein Gag and is required for RNA packaging [162] (Figure 3A).

In sharp contrast with what was described for HIV-1, we showed that HIV-2 replication induces the spontaneous assembly of stress granules [166] (Figure 3B). Moreover, we observed that HIV-2 unspliced mRNA was directly associated with the stress granule assembly factor TIAR in order to form a specific viral mRNP [166] (Figure 3B). We have previously shown that ribosome recruitment onto the HIV-2 genomic RNA is very inefficient due to a strong interference imposed by the highly structured TAR RNA motif [109]. Thus, stress granules could serve as sites of storage for the viral genome while threshold levels of Gag required for RNA packaging are produced. Interestingly, the HIV-2 Gag polyprotein was also observed in stress granules indicating that the transition from translation to RNA packaging may occur in these structures [166] (Figure 3B).

## 8. Viral Proteins Promoting Translation

**Figure 4:**
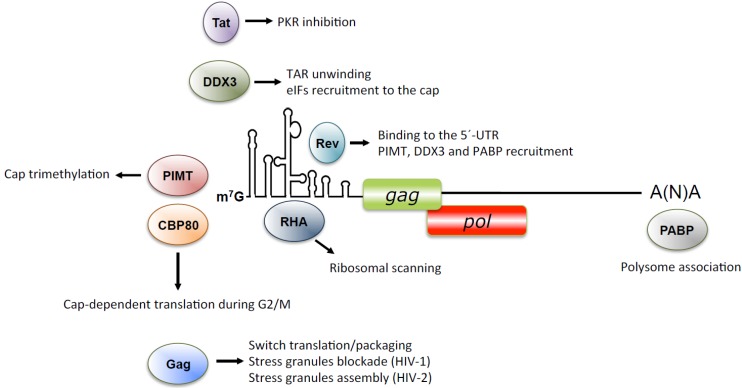
Translational control by host and viral proteins. Schematic representation of the panel of viral (Rev, Tat, Gag) and cellular (DDX3, PIMT, CBP80, RHA, and PABP) proteins required to assist translation initiation from the HIV-1 and HIV-2 unspliced mRNA.

Some of the virally encoded proteins, namely Tat, Rev, and Gag have been involved in the control of viral mRNA translation (Figure 4). Initial studies carried out in the RRL and *Xenopus leavis* oocytes revealed that Tat was involved in the control of translation notably by counteracting the deleterious activation of PKR [107,167,168]. Indeed, secondary RNA structures constituting the TAR motif at the 5′-UTR were shown to activate the protein kinase R (PKR) leading to inhibition of translation [169,170]. Once activated, PKR phosphorylates the α subunit of eIF2 resulting in a global inhibition of translation initiation [171]. However, Tat binding to TAR and/or PKR could prevent activation of the kinase the phosphorylation of eIF2α [172]. In addition, it was shown that Tat is able to stimulate translation both *in vitro* and in living cells [129]. Moreover, binding of Tat to the 5′-UTR of the unspliced mRNA could stimulate the programmed-1 ribosomal frameshift [173]. More recently, Tat was shown to interact with DDX3 and remain associated to polysomes together with the unspliced mRNA further indicating its role in viral mRNA translation [151].

Another viral protein, Rev, was demonstrated to be required for association of the incompletely spliced mRNAs *vif*, *vpr*, *env,* and *vpu* into polysomes [174]. By using a Gag expression vector lacking the RRE, it was also shown that polysome association of the resulting gag mRNA was deficient either in the presence or absence of Rev, suggesting that the Rev-RRE interaction and not the presence of Rev *per se* is critical for ribosome recruitment [175]. Such a function of Rev in translation could be explained by an enhanced recruitment of PABPC1 to Rev-dependent mRNAs [176] or by direct binding to the loop-A of stem-loop 1 located within the packaging signal [177].

Finally the Gag protein was shown to modulate its own translation by exerting a bimodal effect depending on its concentration [143,166,178]. As such, it was shown that HIV-1 and HIV-2 Gag stimulate translation at low concentrations to then inhibit protein synthesis. In the case of HIV-1, stimulation required the matrix domain while inhibition was dependent on the binding of nucleocapsid domain to the packaging signal [178]. In the case of HIV-2, we also observed a bimodal effect of Gag on translation with stimulation at low concentration and inhibition and higher concentrations [166]. Interestingly, we observed that such an effect of Gag on translation was concordant with the subcellular localization of the unspliced mRNA [166]. As such, we observed that both the unspliced mRNA and Gag localized diffusely in the cytoplasm at low concentrations of Gag while both components were assembled in stress granules at high concentrations of the viral protein [166].

## 9. Concluding Remarks

Post-transcriptional control of HIV-1 viral gene expression is regulated from the nucleus to the cytoplasm and involves many host and viral proteins throughout this process. It is amazing to realize that almost every step, from splicing, export, and translation, has its own regulatory pathway, which often differs from that used by cellular mRNAs. This results in the constitution of a unique viral RNP that reaches the cytoplasm to be translated by a spectrum of different mechanisms juggling with cap-dependent and cap-independent mechanisms of initiation. The great diversity of these means of expression confers to the virus several selective advantages such as preventing degradation of the unspliced mRNA by surveillance mechanisms in the nucleus and allowing selective translation under conditions that are not favorable for host gene expression. Evolution of diverse mechanisms for gene expression also allows the conciliation of the presence of multiple RNA structures in the 5′-UTR, that are required for genome replication, with the need of an efficient mechanism for viral protein synthesis. For instance, the TAR structure at the 5′ end of the mRNA represents an essential element for transcription that would be severely inhibitory for ribosome binding and scanning unless it can be counteracted by the recruitment of the host RNA helicase DDX3 to assist pre-initiation complex formation. An interesting, promising new direction concerns the recent identification of compartmentalized cytoplasmic foci containing HIV-1 and HIV-2 viral RNPs. Although, the function of these foci are not fully characterized, they may serve as sites of storage to ensure an equilibrium between unspliced mRNA translation and its packaging into assembly virions. Interestingly, use of these cytoplasmic foci seems to be radically different between the two closely related human immunodeficiency viruses. A better understanding of this process may shed light on our understanding of the replication cycle of these two relatives. Such a specific and complex control of post-transcription gene expression in lentiviruses can point to new directions in the treatment of disease. As such, the targeting of essential host factors that are required for viral replication, such as DDX3 for instance, could bring new therapeutical approaches. Above all, due to great diversity of their strategies developed to express their genome, lentiviruses represent good paradigms for the studies on the control of post-transcriptional gene expression.
